# Use and limitations of malaria rapid diagnostic testing by community health workers in war-torn Democratic Republic of Congo

**DOI:** 10.1186/1475-2875-8-308

**Published:** 2009-12-23

**Authors:** Michael Hawkes, Jean Paul Katsuva, Claude K Masumbuko

**Affiliations:** 1 University of Toronto, 101 College St, Suite 10-401, Toronto, Ontario, Canada; 2 Hospital for Sick Children, Toronto, Canada; 3 Institut Supérieur des Techniques Médicales de Kisangani, Kisangani, Democratic Republic of Congo; 4 HEAL Africa, Goma, Democratic Republic of Congo

## Abstract

**Background:**

Accurate and practical malaria diagnostics, such as immunochromatographic rapid diagnostic tests (RDTs), have the potential to avert unnecessary treatments and save lives. Volunteer community health workers (CHWs) represent a potentially valuable human resource for expanding this technology to where it is most needed, remote rural communities in sub-Saharan Africa with limited health facilities and personnel. This study reports on a training programme for CHWs to incorporate RDTs into their management strategy for febrile children in the Democratic Republic of Congo, a tropical African setting ravaged by human conflict.

**Methods:**

Prospective cohort study, satisfaction questionnaire and decision analysis.

**Results:**

Twelve CHWs were trained to safely and accurately perform and interpret RDTs, then successfully implemented rapid diagnostic testing in their remote community in a cohort of 357 febrile children. CHWs were uniformly positive in evaluating RDTs for their utility and ease of use. However, high malaria prevalence in this cohort (93% by RDTs, 88% by light microscopy) limited the cost-effectiveness of RDTs compared to presumptive treatment of all febrile children, as evidenced by findings from a simplified decision analysis.

**Conclusions:**

CHWs can safely and effectively use RDTs in their management of febrile children; however, cost-effectiveness of RDTs is limited in zones of high malaria prevalence.

## Background

Malaria remains the leading parasitic cause of morbidity and mortality worldwide, causing an estimated 515 million clinical cases and 2 million deaths annually[1]. Children in sub-Saharan Africa carry the highest burden of illness, accounting for 75% of all fatal cases[2]. The management of malaria in resource-poor areas is complicated by the lack of trained health professionals, the non-specific presentation of malaria (which may be indistinguishable clinically from benign viral illnesses or life threatening bacterial infections), and the lack of accessible and affordable diagnostic tools. Conflict and human insecurity further amplify these challenges through a breakdown in health delivery systems, loss of human resources for health, and increase in the prevalence of infectious diseases such as malaria[3,4].

Accurate malaria diagnosis remains a cornerstone of global control efforts. However, several authors have noted that malaria misdiagnosis in endemic areas is common, resulting in harm to vulnerable populations[5]. It is estimated that practical and accurate diagnostic tests for malaria diagnosis have the potential to avert 400 million unnecessary treatments and save 100,000 lives annually[6]. Lateral flow immunochromatographic devices, or rapid diagnostic tests (RDTs), offer the possibility of sensitive and specific field parasitological diagnosis of malaria through the detection of parasite antigen, such as histidine-rich protein 2[7,8]. However, operational questions remain as to how best to distribute and utilize RDTs, particularly in areas where the burden of malaria exceeds the health system capacity for malaria management.

In this setting, community health workers may represent a valuable human resource for the case management of malaria. Previous reports from South America[9,10], Asia[11] and Africa [12-14] have described the successful mobilization of community health workers to diagnose and treat malaria in remote villages using RDTs. In Cambodia, for example, village malaria workers have provided accessible malaria diagnostic and treatment services in remote communities since 2001, in a programme which has been scaled-up to involve over 300 villages[11]. Pushing the use of RDTs to the periphery is an important priority if this technology is to benefit rural populations[6]; however, local conditions, such as malaria prevalence and pre-existing infrastructure, need to be considered for appropriate roll-out of this technology.

As a promising strategy for improving capacity for malaria management, the use of RDTs by CHWs in a rural setting in eastern Democratic Republic of Congo was investigated. This malaria holoendemic area has been crippled by civil and international war and currently has limited health personnel and facilities. This study reports the outcome of a training module for community health workers, and demonstrates the feasibility of RDT use by community health workers in a cohort of 357 febrile children in their remote village. However, the findings of this study, supported by a decision analysis using locally relevant cost parameters, call into question the cost-benefit of rapid diagnostic tests in a zone of malaria holoendemicity.

## Methods

### Study area and population

Over the past decade, the Democratic Republic of Congo has been the stage of the most deadly humanitarian crisis in recent history[3,4]. Despite an official end to the conflict, mortality rates remain 70% higher than pre-war levels and 55% higher than surrounding sub-Saharan African countries[3]. Children under five years of age account for nearly half of all deaths[3]. Most deaths are due to indirect effects of human insecurity (e.g., displaced persons and degradation of health care infrastructure) and fever/malaria is the leading cause of mortality[3]. The region of Yakusu in Orientale province is a remote community of 172,390 inhabitants, with only four doctors. *Plasmodium falciparum *accounts for more than 95% of clinical cases of malaria, and children may experience an estimated three to four clinical episodes of malaria annually.

In this underserved area, community health workers form an integral part of the health care system. They are volunteers that have chosen to devote some of their time to manage health problems in their community, acting as a bridge between the community and the government health care system. They are chosen by community members and contribute to the sustainable development of the health system at the village level. They have minimal (typically <1 week) training in epidemic and endemic diseases (e.g., malaria), generally provided by non-governmental organizations. Although they do not receive a salary from the central government, CHWs may derive a profit (e.g., 10% of insecticide-treated bed net sales) from their activities. The CHWs in this study had previous experience in the management of febrile children in their community. The goal of this study was to build on their existing capacities and extend their diagnostic armamentarium with RDTs.

Ethics approval for this study was obtained by the local Congolese university institution (Institut Supérieur des Techniques Médicales de Kisangani).

### Training

Twelve experienced health care workers from Yakusu village were selected to attend a one-day training session on malaria, its clinical manifestations, treatment, and the use of RDTs for parasitological diagnosis. The RDT in this study was the commercially available Paracheck-Pf^(R) ^kit (Orchid Biomedical Systems, Goa, India). One instructor (JPK) taught and evaluated CHWs. Tools used for teaching included materials published by the WHO[15], including pictorial teaching aids developed elsewhere[13]. Teaching methods included lecture-style sessions (malaria biology and clinical case management) and hands-on demonstration of how to use the RDT. Novel and published instruments for the evaluation of health care worker knowledge and skills at performing and interpreting the RDT were used[15]. A 10-item written multiple choice quiz testing knowledge of the causes and management of fever and malaria in children was administered at the end of the day. In addition, practical skills were evaluated using a standard, objective 16-item WHO RDT training evaluation instrument[15]. Participants were individually and directly observed while performing a RDT, and graded using this checklist.

### Field implementation

Following training and verification of knowledge and skills in the safe and reliable use of RDTs, CHWs were distributed thermometers, RDTs and malaria treatment (artemisinin combination therapy, ACT) for use upon return to their community. Interpretation of the malaria RDT was positive if both the control and test lines were present and negative if only the control line was present. In cases where the control line did not appear, the result was considered indeterminate. Children (0 to 14 years old) with fever (>38°C axillary temperature) were eligible to be tested with RDTs and were treated with ACT (artesunate + amodiaquine, fixed dose combination) dosed by weight, according to WHO guidelines[16]. Participant caregivers provided verbal consent to participate in the study, and were offered free treatment as a benefit of study participation. All febrile children were treated, regardless of RDT result, given the investigational nature of this study, for maximal safety of participants and consistent with local practices. Febrile children of all ages were included, as the purpose of the study was to evaluate the feasibility of RDT use by CHWs, although WHO treatment guidelines recommend treatment of all febrile children under five years of age, irrespective of RDT result[16]. Children requiring more extensive workup or interventions were referred to regional health services; the decision to refer was left to the discretion of CHWs, who were previously experienced in malaria management. For each child, a case report form was completed, including demographic information, temperature, other signs and symptoms, RDT result, and clinical outcome. Results were collected and tabulated centrally. Because a high prevalence of test positivity was noted in this sample of febrile children, peripheral blood smears were collected and examined by light microscopy to confirm this observation in an additional sample of 40 children with fever in the same community. Descriptive statistics were calculated using Microsoft Excel software.

### Survey of CHW views following field trial of RDTs

After the field trial of RDTs, CHWs were assembled and surveyed using a 16-item written survey questionnaire. Questions consisted of provocative statements with which participants ranked their agreement or disagreement on a 5-point Likert scale.

### Decision analysis

To examine the cost-effectiveness of malaria RDTs in this setting, a decision analysis tool was used, modified from previous studies[17]. The goal was to investigate the cost associated with RDTs, given that they prevent unnecessary treatment courses and, therefore, several simplifying assumptions were made. The cost of false-negative diagnosis was neglected, given that only 1.1% of the cohort tested negative using RDT. The potential benefit of RDTs in alerting clinicians to the possibility of other causes of fever besides malaria was also neglected, given the limited resources for management of other infections in this particular setting. This approach and these assumptions were similar to some previous cost analyses[18], but less detailed than other published models[17]. The simplicity of this model permitted derivation of an analytic equation of the cost per unnecessary treatment averted as a function of disease prevalence, costs, and test sensitivity and specificity. Inputs for the model included published estimates of RDT sensitivity and specificity[8], and the actual local cost of RDTs including lancets, alcohol swabs, gloves and test kits ($2) and ACT ($1 per paediatric treatment course).

## Results

Twelve community health workers were trained to perform and interpret malaria RDTs (Paracheck-Pf^(R)^). Median health worker age was 32 (range 23 to 47) years, and 4/12 were female. Participants had a median of 4.5 (range 1 to 10) years previous health care experience, all had previously managed cases of malaria, but only 2/12 had used a malaria RDT previously. Participants were highly satisfied with the training. Eleven of 12 agreed or strongly agreed that the information provided was useful; and although 7/12 strongly agreed that they already knew about malaria management beforehand, 11/12 agreed or strongly agreed that their knowledge had improved as a result of the training session. Qualitative feedback emphasized the comprehensiveness, clarity and quality of the teaching materials and trainer.

Participants demonstrated satisfactory knowledge and skills following the training session. Median (range) scores on a 10-item test of the causes and management of fever and malaria in children were 80% (60% to 90%). Questions pertaining to the causes of fever in children and the clinical signs of malaria were answered correctly by all participants. Poorer performance was observed on questions involving clinical scenarios: for example, 5/12 participants incorrectly answered that a febrile well-appearing 10 year old child with a negative RDT result should receive anti-malarial treatment. Median (range) scores on a standard WHO 16-item objective RDT skills assessment[15] were 100% (94% to 100%) and all participants correctly interpreted the RDT result during the evaluation.

The 12 trained health workers used the RDTs to manage 357 children with fever in their remote community over a one-week period in February, 2009 (during rainy season). Each health worker managed a median of 4 (range 0 to 9) febrile children each day, which included the administration of a fingerprick malaria RDT. The median age of the children was four years (range 1 month to 13 years), and 51% were less than five years old. The mean axillary temperature of children was 39.3 (standard deviation ± 0.8)°C. Associated signs and symptoms are shown in Table 1 according to patient age; non-specific symptoms of headache, fatigue, chills and poor feeding were most common. Ninety-three percent of children had a positive test result on the malaria RDT, 1.1% were negative and 5.0% were indeterminate. All children were treated with ACT, dosed according to their age[16]. Fever and associated signs and symptoms improved in the majority (96%) of children; however, 15 children (4.2%) required transfer to regional health centres for further investigations or treatment (usually blood transfusion), and one two-month old infant who presented with fever, inconsolable crying, refusal to breastfeed, and a positive RDT, died despite administration of oral ACT.

**Table 1:** Signs and symptoms of febrile children (n = 357)

	Age category		
**Sign or symptom**	**<1 yr (n = 78)**	**1-4 yr (n = 106)**	**≥ 5 yr** **(n = 173)**

Fever	78 (100)	106 (100)	173 (100)

headache	3 (3)	45 (42)	123 (71)

fatigue/lethargy	24 (30)	44 (41)	69 (39)

chills	21 (26)	29 (27)	35 (20)

decreased oral intake/poor feeding	18 (23)	34 (32)	33 (19)

myalgia	0	10 (9)	34 (19)

vomiting	7 (8)	20 (18)	17 (9)

prolonged crying	30 (38)	8 (7)	0

seizures	22 (28)	5 (4)	0

dizziness	0	5 (4)	21 (12)

coryza	2 (2)	8 (7)	17 (9)

cough	3 (3)	5 (4)	9 (5)

pallor	9 (11)	4 (3)	2 (1)

diarrhoea	3 (3)	10 (9)	0

respiratory distress	5 (6)	4 (3)	1 (0.6)

visual disturbance	0	0	3 (1)

splenomegaly	0	0	1 (0.6)

lumbar pain	0	1 (0.9)	2 (1)

abdominal pain	0	0	1 (0.6)

joint pain	0	0	1 (0.6)

Given the high rate of RDT positivity among febrile children in this community, an alternative diagnostic modality (light microscopy) was used to verify the malaria prevalence. An experienced microscopist prospectively examined Giemsa-stained peripheral blood smears by light microscopy in an additional sample of 40 children with fever in the same community. The prevalence of parasitaemia among febrile children was confirmed to be high (88%; 95%CI 74-95%) using this gold-standard diagnostic modality.

Following the trial period of RDTs, CHWs were surveyed to determine their views regarding their experience using RDTs in the field. As a rule, responses were uniform across the 12 health workers. Ten of 12 respondents agreed or strongly agreed that fever was an important clinical problem among children in their community, and all 12 agreed or strongly agreed that the febrile child poses a diagnostic dilemma for which RDTs are useful. All agreed or strongly agreed that RDTs were easy to learn, easy to perform, useful in the management of febrile children, and easy to interpret. All 12 agreed or strongly agreed that the initial training course had been useful. All noted that RDTs were not readily available in their community, 11/12 felt that RDTs would be affordable, and all 12 felt that the government should supply RDTs. Finally, all agreed or strongly agreed that they would continue to use RDTs after the study was finished.

**Figure 1:**
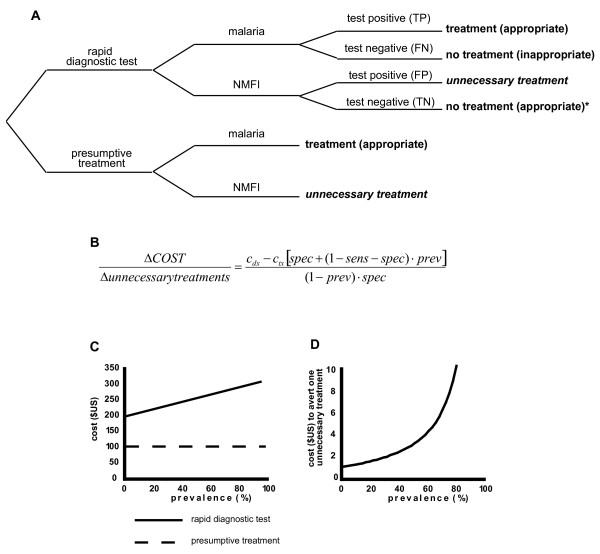
**A simple decision analysis demonstrated elevated cost/utility ratio of rapid diagnostic testing (RDT) strategy at high malaria prevalence, in a hypothetical cohort of 100 febrile children**. A. Decision analysis tree. Among simplifying assumptions (*), the cost of false negative malaria tests is neglected (given high test specificity and high disease prevalence). B. Equation relating the trade-off of increased cost but reduced number of unnecessary treatment courses associated with a strategy of RDT. C. Cost of two strategies (RDT, solid line versus presumptive treatment, dashed line) over a range of malaria prevalence. D. The cost of averting one unnecessary treatment with RDT rises steeply at high prevalence. NMFI, non-malaria febrile illness; TP, true positive; TN, true negative; FP, false positive; FN, false negative; c_dx_, cost of rapid diagnostic test; c_tx_, cost of treatment course; sens, sensitivity; spec, specificity; prev, prevalence.

Next, a simplified decision analysis was employed to examine the cost-benefit of using RDT in a context of high malaria endemicity. Comparison was made between the scenario of universal treatment of all febrile children to the alternative scenario of treating according to RDT result, thereby withholding anti-malarial treatment from children five years and older with a negative RDT (Figure 1). The cost of presumptive treatment and testing over a range of malaria prevalence was evaluated (Figure 1). At a prevalence of 80%, use of RDT was associated with a cost of US$8.79 per unnecessary course of anti-malarial therapy averted (Figure 1).

## Discussion

This study demonstrates the feasibility of training community health workers in the use of malaria RDTs in a setting of high malaria endemicity with scarce human and technological resources for health, exacerbated by violent conflict and human insecurity. In a single day, community health workers were trained to use and interpret a commercially available RDT, demonstrating appropriate knowledge and skills at the end of the training session. They subsequently demonstrated competence in point-of-care parasitological diagnosis of malaria in a group of 357 febrile children under field conditions. Following the field implementation of rapid diagnostic testing, CHWs were uniformly positive about the utility and ease of use of RDTs in their setting. On the other hand, cost remains a major barrier to the use of RDTs in areas of high malaria prevalence, as demonstrated by a simple decision analysis.

This study is noteworthy for its setting in a zone of ongoing human insecurity. Over more than a decade, a civil and international conflagration has claimed over 3.9 million lives in the DRC, more than any other conflict since World War II[4]. Despite an official end to the fighting, Mai Mai rebel insurgents continue to destabilize areas in the east of the country and the current mortality rate remains well above pre-war levels and above levels in surrounding sub-Saharan Africa[3]. Most deaths are not due to the direct effects of violence, but due to preventable, treatable infectious diseases, including malaria, which is the primary cause of mortality in the DRC[3]. In this context, CHWs may play an especially important role in delivering health care to abandoned and displaced populations with little access to formal health services. Relative to previous reports on the use of RDTs by CHWs, the present study goes beyond evaluation of CHW training[13] and description of treatment programmes[11,14]. This study reports on the implementation of rapid diagnostic testing by CHWs in a cohort of febrile children under field conditions in a remote village, along with a survey of CHWs views and attitudes toward RDTs after this practical trial period. Moreover, this study highlights important obstacles to the use of RDTs in practice in this setting, particularly the cost-effectiveness in a zone of high prevalence.

The principal barrier to the utilization of RDTs in community-based malaria care is the sustainable purchase and supply of RDTs. In the decision analysis presented, health service payers would have to value the cost of saving one unnecessary treatment at $8.79, which represents eight times the cost of an ACT treatment course, and 60% of the annual *per capita *public expenditure on health care ($15/yr)[19]. This cost is unlikely to be acceptable to public health care administrators or patient families. Previously, detailed decision analyses have been published which support the finding that RDTs are not cost effective in settings of high malaria prevalence[17]. In one recent report, the probability of RDTs being cost effective is less than 50% beyond a prevalence of 80%, as observed in the present study[17]. Likewise, RDTs were cost-saving only below a prevalence threshold of 52-55% in other analyses[18,20]. Few of these reports consider the implications of their models at high malaria prevalence, although the findings demonstrate the relevance of this situation in tropical Africa. Thus, despite optimism for the utility of RDTs in resource-limited endemic settings, at high malaria prevalence, presumptive treatment appears to be a more acceptable management strategy, based on the elevated cost/utility ratio of RDTs.

As in this cohort of febrile children in tropical Congo, previous studies have documented a high prevalence of parasitaemia among children with fever in holoendemic zones. Test positivity among febrile children in excess of 80% has been documented following a flood disaster in Mozambique (81%)[21] and in the DRC (82%)[22]. Furthermore, it is well-recognized that test positivity persists after parasite clearance for four weeks or more [22-24], such that children with infection in the past month will also test positive. Light microscopy of Giemsa-stained peripheral smears was used to validate this finding and confirmed a high (88%) rate of parasitaemia among febrile children in this area of the DRC. Associated signs and symptoms were non-specific (Table 1), yet consistent with a diagnosis of malaria (e.g., headache, fatigue, chills, and poor feeding). On the other hand, in some cases the diagnosis of malaria may have been incidental, given the likelihood of other common causes of childhood fever such as viral upper respiratory tract infection along with compatible symptoms in this cohort (e.g., coryza). This highlights a more general need for diagnostic tools appropriate to the rural African context for infectious diseases beyond malaria alone. In addition to cost, other impediments to the widespread use of RDTs by CHWs include concerns about transmission of blood-borne infections including HIV. However, this and other studies[13] have demonstrated that CHWs can be trained to safely perform blood sampling for the RDT. Gloves, lancets and proper disposal should be incorporated into training modules and factored into the cost of RDT programmes. Variation in test sensitivity due to damage during transportation and/or use at high temperature or humidity[7] constitutes another challenge to the reliable use of RDTs in remote communities, highlighting the need for quality control measures (e.g., microscopy confirmation of negative test results), although this strategy poses challenges of its own in severely resource-restricted areas. Acceptability of RDTs versus microscopy or clinical diagnosis may represent an additional barrier, although CHWs were uniformly positive about the utility of RDTs when surveyed following the field trial.

Although RDTs appear to be prohibitively expensive in this context, the mobilization of CHWs for malaria management and control remains a relevant strategy. In this study, 12 highly motivated CHWs who had previous experience managing children with malaria (although only two had previously used a RDT), demonstrated efficient integration of knowledge and skills in malaria diagnosis and treatment, and were rapidly mobilized to treat a large number of febrile children in a remote setting. Access to government sponsored health services may be as low as 50% in this and similar areas of sub-Saharan Africa[5], emphasizing the need for community based interventions. CHWs have proven valuable in other geographic areas such as Cambodia, where a national programme of malaria health workers currently provide accessible malaria diagnosis and treatment in over 300 villages[11]. In the DRC, where violent conflict has severely disrupted government health infrastructure and has led to the displacement of large numbers of people in a zone of high malaria transmission, CHWs may represent a valuable human resource to address the intolerable burden of malaria, currently the number one cause of childhood mortality[3].

Limitations of this study include the relatively small sample size of CHWs from a single geographic area, and the short time span (one week) during which children were diagnosed and treated for malaria. Although a study involving multiple sites over a prolonged period might provide more generalizable information, these results nonetheless underline some important considerations for the roll-out of RDTs in other areas, particularly the prevalence of malaria among the target population for testing. During dry season, or in areas where malaria prevalence is lower, RDTs may be more cost-effective than determined in this study. With respect to survey questionnaires on the utility of the training session and the acceptability of RDTs, we cannot exclude the possibility of "social courtesy bias" influencing CHW to give positive feedback, although surveys were conducted anonymously to minimize this potential bias. Despite the lack of formal medical or nursing training, the CHWs in this study were relatively experienced in malaria management, which may explain their ability to rapidly incorporate RDTs into their clinical practice. Thus, successes in training CHWs in this context should not necessarily be extrapolated to less experienced personnel. RDT test performance characteristics (sensitivity and specificity) were not assessed against a gold standard, because this has been extensively documented in previous studies (reviewed in [8]).

In summary, this study demonstrates the feasibility of training CHWs in the use of RDTs for malaria diagnosis and management among febrile children in a remote community affected by violent conflict. This study illustrates the importance of considering local factors (e.g., malaria prevalence) in assessing the appropriateness of RDTs for remote and vulnerable populations.

## Competing interests

The authors declare that they have no competing interests

## Authors' contributions

MH conceived the study, performed the analysis, and wrote the manuscript. JPK trained the CHWs, supervised the field study of RDTs, and collected the data. CKM provided intellectual input into study design and implementation, and revised the manuscript. All authors have read and approved the final manuscript.

## References

[B1] Snow RW Guerra CA Noor AM Myint HY Hay SI The global distribution of clinical episodes of *Plasmodium falciparum *malaria Nature 2005 434 214 217 10.1038/nature03342 15759000 PMC3128492

[B2] World Health Organization Global Malaria Programme http://www.who.int/malaria

[B3] Coghlan B Ngoy P Mulumba F Hardy C Bemo VN Stewart T Lewis J Brennan RJ Update on mortality in the Democratic Republic of Congo: results from a third nationwide survey Disaster Med Public Health Prep 2009 3 88 96 10.1097/DMP.0b013e3181a6e952 19491603

[B4] Coghlan B Brennan RJ Ngoy P Dofara D Otto B Clements M Stewart T Mortality in the Democratic Republic of Congo: a nationwide survey Lancet 2006 367 44 51 10.1016/S0140-6736(06)67923-3 16399152

[B5] Amexo M Tolhurst R Barnish G Bates I Malaria misdiagnosis: effects on the poor and vulnerable Lancet 2004 364 1896 1898 10.1016/S0140-6736(04)17446-1 15555670

[B6] Rafael ME Taylor T Magill A Lim YW Girosi F Allan R Reducing the burden of childhood malaria in Africa: the role of improved diagnostics Nature 2006 444 Suppl 1 39 48 10.1038/nature05445 17159893

[B7] Hawkes M Kain KC Advances in malaria diagnosis Expert Rev Anti Infect Ther 2007 5 485 495 10.1586/14787210.5.3.485 17547512

[B8] Ochola LB Vounatsou P Smith T Mabaso ML Newton CR The reliability of diagnostic techniques in the diagnosis and management of malaria in the absence of a gold standard Lancet Infect Dis 2006 6 582 588 10.1016/S1473-3099(06)70579-5 16931409

[B9] Cunha ML Piovesan-Alves F Pang LW Community-based program for malaria case management in the Brazilian Amazon Am J Trop Med Hyg 2001 65 872 876 11791990 10.4269/ajtmh.2001.65.872

[B10] Pang LW Piovesan-Alves F Economic advantage of a community-based malaria management program in the Brazilian Amazon Am J Trop Med Hyg 2001 65 883 886 11791992 10.4269/ajtmh.2001.65.883

[B11] Yeung S Van Damme W Socheat D White NJ Mills A Access to artemisinin combination therapy for malaria in remote areas of Cambodia Malar J 2008 7 96 10.1186/1475-2875-7-96 18510724 PMC2430580

[B12] Premji Z Minjas JN Shiff CJ Laboratory diagnosis of malaria by village health workers using the rapid manual ParaSight-F test Trans R Soc Trop Med Hyg 1994 88 418 10.1016/0035-9203(94)90409-X 7570824

[B13] Harvey SA Jennings L Chinyama M Masaninga F Mulholland K Bell DR Improving community health worker use of malaria rapid diagnostic tests in Zambia: package instructions, job aid and job aid-plus-training Malar J 2008 7 160 10.1186/1475-2875-7-160 18718028 PMC2547110

[B14] Elmardi KA Malik EM Abdelgadir T Ali SH Elsyed AH Mudather MA Elhassan AH Adam I Feasibility and acceptability of home-based management of malaria strategy adapted to Sudan's conditions using artemisinin-based combination therapy and rapid diagnostic test Malar J 2009 8 39 10.1186/1475-2875-8-39 19272157 PMC2660358

[B15] World Health Organization Matériel de formation pour les TDRs génériques de Falciparum http://www.wpro.who.int/sites/rdt/using_rdts/training

[B16] World Health Organization Guidelines for the Treatment of Malaria 2006 http://whqlibdoc.who.int/publications/2006/9241546948_eng.pdf

[B17] Shillcutt S Morel C Goodman C Coleman P Bell D Whitty CJ Mills A Cost-effectiveness of malaria diagnostic methods in sub-Saharan Africa in an era of combination therapy Bull World Health Organ 2008 86 101 110 10.2471/BLT.07.042259 18297164 PMC2647374

[B18] Zikusooka CM McIntyre D Barnes KI Should countries implementing an artemisinin-based combination malaria treatment policy also introduce rapid diagnostic tests? Malar J 2008 7 176 10.1186/1475-2875-7-176 18793410 PMC2556342

[B19] United Nations Development Programme. 2007/2008 Human development Report. Country Factsheet, Democratic Republic of Congo http://hdrstats.undp.org/en/countries/data_sheets/cty_ds_COD.html

[B20] Rolland E Checchi F Pinoges L Balkan S Guthmann JP Guerin PJ Operational response to malaria epidemics: are rapid diagnostic tests cost-effective? Trop Med Int Health 2006 11 398 408 10.1111/j.1365-3156.2006.01580.x 16553923

[B21] Hashizume M Kondo H Murakami T Kodama M Nakahara S Lucas ME Wakai S Use of rapid diagnostic tests for malaria in an emergency situation after the flood disaster in Mozambique Public Health 2006 120 444 447 10.1016/j.puhe.2005.11.007 16530797

[B22] Swarthout TD Counihan H Senga RK Broek I van den Paracheck-Pf accuracy and recently treated *Plasmodium falciparum *infections: is there a risk of over-diagnosis? Malar J 2007 6 58 10.1186/1475-2875-6-58 17506881 PMC1890550

[B23] Humar A Ohrt C Harrington MA Pillai D Kain KC Parasight F test compared with the polymerase chain reaction and microscopy for the diagnosis of *Plasmodium falciparum *malaria in travelers Am J Trop Med Hyg 1997 56 44 48 9063360 10.4269/ajtmh.1997.56.44

[B24] Huong NM Davis TM Hewitt S Huong NV Uyen TT Nhan DH Cong le D Comparison of three antigen detection methods for diagnosis and therapeutic monitoring of malaria: a field study from southern Vietnam Trop Med Int Health 2002 7 304 308 10.1046/j.1365-3156.2002.00869.x 11952945

